# First Record of Isolation and Characterization of Methicillin Resistant *Staphylococcus lugdunensis* from Clinical Samples in Iraq

**DOI:** 10.1155/2014/736259

**Published:** 2014-07-13

**Authors:** Alaa H. Al-Charrakh, Mohammed H. Obayes

**Affiliations:** Department of Microbiology, College of Medicine, Babylon University, Hilla, Babylon Governorate, Iraq

## Abstract

This study was conducted to determine the frequency of * Staphylococcus lugdunensis* in different clinical samples. Out of 690 clinical samples, a total of 178 coagulase negative staphylococci (CoNS) isolates were recovered. CoNS were identified as 10 different species; 22 isolates belonged to * Staphylococcus lugdunensis*. Two specific genes for * S. lugdunensis *were used ( *tanA *gene and * fbl *gene) to confirm identification. Both of these specific genes were detected in 15 (68.1%) of 22 isolates that were identified phenotypically. The results of oxacillin MIC showed that 7 of the 15 (46.6%) * S. lugdunensis *isolates were oxacillin resistant. The antibiotic susceptibility testing against 16 antibiotics showed that resistance rates were variable towards these antibiotics. Eight of fifteen * S. lugdunensis *isolates (53.3%) were *β*-lactamase producer. Results of molecular detection of * mecA *gene found that * mecA *gene was detected in 6 (40%) of 15 * S. lugdunensis*. All of these 6 isolates (S1, S2, S3, S4, S5, and S6) were resistant to oxacillin. One isolate (S7) was resistant to oxacillin but * mecA *was not detected in this isolate. This study is a first record of isolation and characterization of methicillin resistant *S. lugdunensis *(MRSL) from clinical samples in Iraq.

## 1. Introduction

The coagulase-negative staphylococci (CoNS) is a large group of Gram-positive bacteria most often found colonizing the skin and mucosal surfaces of humans and other mammals [1]. Several species of CoNS are recognized as potential pathogens, mainly causing nosocomial infections, often involved in infections related to implanted medical devices such as intravenous catheters, prosthetic heart valves, and orthopedic implants.* Staphylococcus lugdunensis *is a coagulase-negative staphylococci first described by Freney and his colleagues in 1988 [2]. The organism is found as a skin commensal in healthy individuals.* S. lugdunensis* has been implicated in invasive diseases, especially fulminant native and prosthetic-valve endocarditis [3]. Other invasive infections include brain abscess, meningitis, skin abscesses, soft tissue infections, spondylodiscitis, foreign body infections, and peritonitis [4].

*S. lugdunensis* shares a number of potential virulence factors with* S. aureus.* In particular,* S. lugdunensis* may express a clumping factor and produce a thermostable DNase [5].* S. lugdunensis* produces a tannase (tannin acyl hydrolase) that degrades hydrolysable tannins [6]. The phenotypic biological tests, such as the ornithine decarboxylase test and genotypic molecular tests, have been developed to identify these bacteria [7]. Several nucleic acid targets that permit the differentiation of* S. lugdunensis* from other CoNS have been exploited using molecular methods. These include the 16S rRNA gene, which was used to confirm identity of* S. lugdunensis* isolate [8], and the* rpoB* gene, which is also specific to* S. lugdunensis* [4]. The* tanA* gene that coded tannase acyl hydrolase was detected in* S. lugdunensis* [6]. A fibrinogen-binding protein known as Fbl that encoded by* fbl* gen is specific to* S. lugdunensis* [9]. The* mecA* gene has been reported in several data, the first in a neonate [10] with methicillin resistant* S. lugdunensis* (MRSL) that produces an alternative penicillin binding protein (PBP2A). Although several researchers have been reported phenotypic and molecular characterizations of methicillin resistant* S. lugdunensis* (MRSL) worldwide, no information are available about these resistant bacteria in Iraq. Therefore the main goals of this study were to isolate and determine the antibiotic resistance patterns of these important bacteria from clinical samples and detect the presence of* mecA* gene that encodes methicillin resistance to confirm being MRSL.

## 2. Materials and Methods

### 2.1. Patients and Samples Collection

This study included 630 patients (aged 2 days–70 years) suffering from different infections who were admitted to four health centers in Al-Hilla city, Iraq. These patients were admitted to different hospital wards, in addition to swabs taken from private clinics during a period extending from November 2012 to the end of May 2013.

Swabs of different samples 690 were generally collected from different sites (wound, burn, blood culture, subaxillary, urine, stool, sputum, throat, ear, skin lesion, high vaginal, and other different swabs). Each sample was immediately inoculated on the blood agar plates and mannitol salt agar. The swab has been inoculated on culture media and incubated aerobically for 24 hours at 37°C. Information about age, antibiotic usage, residence, and hospitalization of patients was taken into consideration.

### 2.2. Bacterial Isolates

*Staphylococcus lugdunensis* isolates were recovered and identified based on their morphology, Gram-staining, catalase test, coagulase test, and ornithine decarboxylation test [11]. Identification was confirmed using two specific genes (*tanA* and* fbl* genes) by PCR assay [12].

### 2.3. Screening of *β*-Lactam Resistant Isolates

Fifteen* S. lugdunensis* isolated were subjected to *β*-lactam resistance screening test as a phenotypic selection test. Preliminary screening of* S. lugdunensis* isolates resistance to *β*-lactam antibiotics was carried out by using pick and patch method on Muller-Hinton agar plates supplemented with ampicillin. All of 15* S. lugdunensis* isolates were subjected to oxacillin resistance screening test by using the same method on Muller-Hinton agar plates supplemented with 4% NaCl and oxacillin 6 *μ*g/mL [13].

### 2.4. Antimicrobial Susceptibility Testing

The antimicrobial susceptibility patterns of isolates to different antibiotics were determined using disk diffusion test and interpreted according to CLSI guidelines [13]. The following antibiotics were obtained (from Oxoid, UK, and Himedia, India) as standard reference disks as known potency for laboratory use: ampicillin (10 *μ*g), oxacillin (5 *μ*g), cloxacillin (5 *μ*g), cefoxitin (30 *μ*g), amoxicillin-clavulanate (20/10 *μ*g), cefexime (30 *μ*g), ceftriaxone (30 *μ*g), imipenem (10 *μ*g), azithromycin (15 *μ*g), and doxycycline (30 *μ*g). The susceptibility to ampicillin, oxacillin, and vancomycin was also determined using twofold agar dilution method [14].

### 2.5. Detection of *β*-Lactamase Production

Nitrocefin diagnostic disk (Fluka, Switzerland) was used to detect the ability of 15 isolates to produce *β*-lactamase. A number of required nitrocefin disks were placed into sterile empty Petri dish and moistened with one drop of sterile D.W.; then the disk was holed by sterile forceps and wiped across a young colony on agar plate. The development of a red color in the area of the disk where the culture was applied indicated a positive result.

### 2.6. Detection of* tanA*,* fbl*, and* mecA* Genes

Three genes were detected in present study, first* tanA* gene that coded to tannase acyl hydrolase enzyme that degrades tannin. The second gene was* fbl* gene that coded to fibrinogen binding protein. The third gene that was detected in the present study was* mecA* that was responsible for oxacillin/methicillin resistance by coding for penicillin binding protein (PBP2a); the primer sequence of these genes were (*tanA F*: AGCATGGGCAATAACAGCAGTAA,* tanA R*: GCTGCGCCAATTTGTTCTAAATAT) 239 bp; the conditions were 95°C 3 min 1x, 94°C 20 sec, 60°C 20 sec 25x, 72°C, 20 sec, and 72°C 5 min 1x [12].

For (*fbl F*: GTAAATAGCGAGGCACAAGC,* fbl R*: GGTAAATCGTATCTGCCGCT) 425 bp, the conditions were 94°C 3 min 1x, 94°C 1 min, 60°C 1 min 30x, 72°C 1 min, and 72°C 5 min 1x [15]. For (*mecA F*: TCCAGGAATGCAGAAAGACCAAAGC,* mecA R*: GACACGATAGCCATCTTCATGTTGG) 499 bp, the conditions were 94°C 3 min 1x, 94°C 1.5 min, 55°C 1 min 36x, 72°C 1 min, and with final step 72°C 10 min 1x [16].

### 2.7. Bacteriological Analysis and Antibiotic Susceptibility of* “S. pseudolugdunensis”* Isolates

*“Staphylococcus pseudolugdunensis”* isolates recovered in this study were identified using the same methods as in* S. lugdunensis*. According to [17], any isolate that diagnosed phenotypically as* S. lugdunensis* but it was negative to* tanA* and* fbl* genes was rediagnosed as* “S. pseudolugdunensis*.*”* The antibiogram, *β*-lactamase production, and presence of* mecA* gene for these isolates were also determined as above.

## 3. Results and Discussion

### 3.1. Isolation and Identification of* S. lugdunensis* Isolates

A total of 690 clinical samples were collected; 602 (87.24%) gave positive growth on blood agar medium, while 88 (12.76%) gave no growth. Out of 393 Gram-positive bacteria, 306 (77.8%) were identified as staphylococci based on morphological characteristics and biochemical tests. According to result of coagulase test, the 306 staphylococci isolates were divided into coagulase positive 128 (41.8%) and coagulase negative 178 (58.2%) (Table 1).

Result of present study was similar to that of Bouza and his colleagues [18], who found that bacterial isolates from clinical samples included 70.7% Gram-positive, 22.2% of Gram-negative, and 7.2% of yeast; they also found that* S. aureus* and CoNS constituted 40% and 60%, respectively. In a local study in Iraq, Al-Fuadi [19] found that total of 148 bacterial isolates represented by different Gram-positive and Gram-negative bacteria in percentage of 77% and 23% respectively. He also found that a total of 31 of 100* Staphylococcus* isolates belonged to* S. aureus*. This difference may have belonged to variation of samples collected in this study.

Results also showed that the highest percentages of CoNS in subaxillary swabs and wound swabs were 29.2% and 23.5%, respectively. The prevalence of* S. lugdunensis* was 22 (12.3%), which is higher than results of several researchers. This may be due to the fact that depending on phenotypic characteristics alone is insufficient and may result in misidentification of* S. lugdunensis*. So, the present study depended (in addition to phenotypic characteristics) on the genotypic characteristics (PCR) to confirm the result. Depending on PCR results, out of 22 of CoNS that were identified phenotypically as* S. lugdunensis* isolates, 15 (8%) were identified as* S. lugdunensis*, while the other seven isolates were belonged to “*S. pseudolugdunensis*.”

Clinical isolates were as follows: subauxiliary swab (4), skin swabs (2), wound (3), burn (1), blood (1), throat swab (2), ear swab (1), and peritonitis (1), while no* S. lugdunensis* isolates were recovered from urine, sputum swabs, stool swabs, and high vaginal swabs. Skin swabs represented folliculitis, boils, and abscesses. Koksal and his colleagues [20] found that it constituted 9% of CoNS isolates from blood culture, while other researchers found that* S. lugdunensis* constituted only 3.3% of CoNS collected from different samples [21].

Results revealed that the seven* “S. pseudolugdunensis”* isolates were recovered from clinical samples of skin (1), urine (1), burn swabs (2), subauxiliary swabs (2), and acne (1).

### 3.2. Molecular Characterization of* S. lugdunensis* Isolates

Definite phenotypic identification of a Gram-positive, catalase-positive coccus as* S. lugdunensis *implies a negative tube coagulase test and positive ornithine decarboxylase activities [22]. However, complete hemolytic, yellow pigmentation, and detection of a fibrinogen affinity factor, although not consistently expressed by* S. lugdunensis, *may leadto its misidentification as* S. aureus *[1].* S. lugdunensis *is an unusually virulent coagulase-negative species, associated with severe infection. So, using single-step, species-specific PCR protocol for* S. lugdunensis *identification is very important [15].

#### 3.2.1. Detection of* tanA *Gene

**Figure 1:**
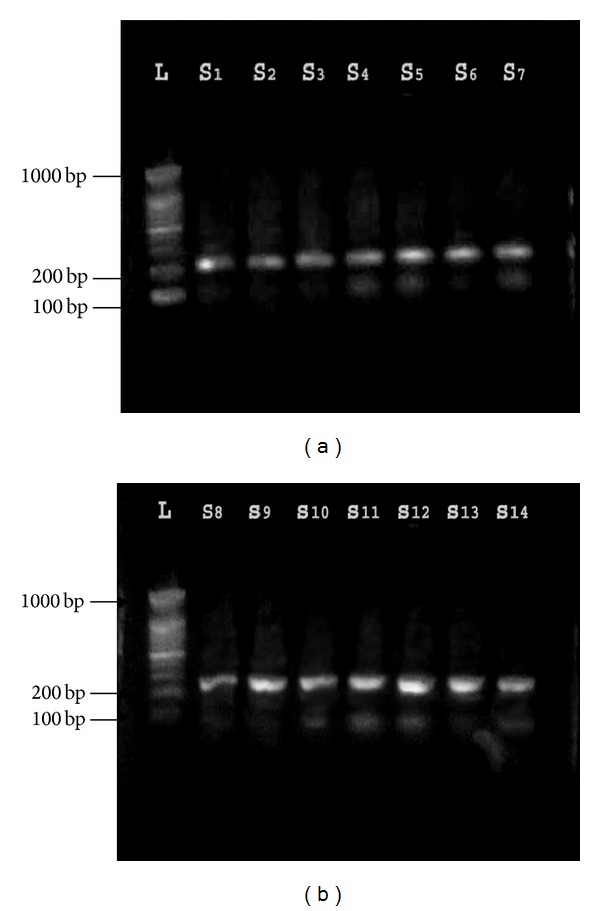
Gel electrophoresis of PCR of* tanA* amplicon (239 bp) product: Lane L: ladder (1000-bp ladder); Lanes S1, 2, 3, 4, 5, 6, 7, 8, 9, 10, 11, 12, 13, and 14: number of* S. lugdunensis* isolates from different clinical samples.

The specific* tanA *gene for* S. lugdunensis *was detected in 15 (68.1%) of 22 isolates that were identified phenotypically. These 15 isolates were identified as* S. lugdunensis *(Figure 1). The remaining 7 isolates (31.9%) were reidentified as* “S. pseudolugdunensis”* [17]. Result also found that* S. aureus *and* S. epidermidis *that were used as negative control had no* tanA* gene which confirms the result of Noguchi and his coworkers [12] who found that no gene or protein homologous to* tanA* were found in a similarity search using published databases such as Gen Bank. These results strongly suggest that* tanA *is specific to* S. lugdunensis*.

#### 3.2.2. Detection of* fbl *Gene of* S. lugdunensis *Isolates

**Figure 2:**
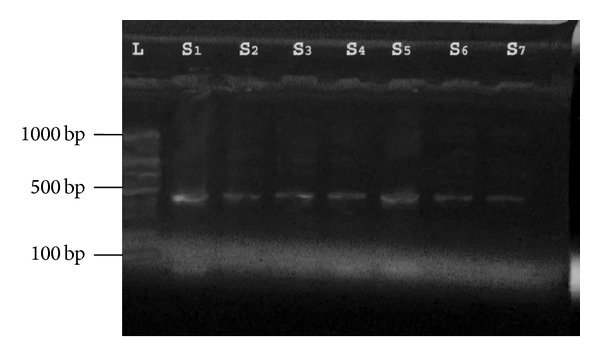
Gel electrophoresis of PCR of* fbl* amplicon (425 bp) product: Lane L: ladder (1000-bp ladder); Lanes S1, 2, 3, 4, 5, 6, and 7: number of* S. lugdunensis* isolates from different clinical samples.

**Figure 3:**
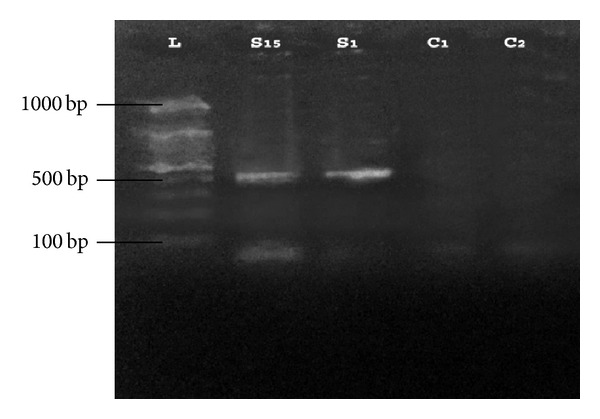
Gel electrophoresis of PCR of* fbl* amplicon (425 bp) product: Lane L: ladder (1000-bp ladder); Lanes S1, and 15: number of* S. lugdunensis* isolates. C1:* S. aureus*, C2:* S. epidermidis*.

A suitable nucleic acid target to diagnosed* S. lugdunensis *is* fbl *gene, which encods a fibrinogen-binding adhesin [15]. The gene was detected in all 15* S. lugdunensis* isolates that were positive to* tanA *in this study (Figure 2), while no amplification product was obtained from* S. aureus *and* S. epidermidis *isolates that were used as negative control as in Figure 3.

According to results of PCR results, among 22* S. lugdunensis *that diagnosed phenotypically, 15 isolates were found to be positive to* tanA *and* fbl *genes that were specific to* S. lugdunensis* [4], so other isolates (number = 7) were diagnosed as* “S. pseudolugdunensis”* [17].

### 3.3. Primary Screening of *β*-Lactam Resistant Isolates

The results of screening test showed that 11 isolates (73.3%) of* S. lugdunensis *were resistant to ampicillin. All these isolates were able to grow normally in the presence of ampicillin; this may be attributed to most of* S. lugdunensis* isolates (about 90% of them) that are coming from several infectious sources (nosocomial infections and other anatomical sites) that are resistant to penicillin [23] due to production of *β*-lactamases that acts in the hydrolysis of *β*-lactam ring of penicillin which is transformed into acid neutralizing its bactericidal effect [23].

The results of oxacillin resistant screening test showed that 7 of the 11 (63.6%) *β*-lactam resistant* S. lugdunensis *isolates were oxacillin resistant. This result was in concordance with study of Mateo and her coworkers [24] who referred to identifying methicillin resistance using the Vitek 2 system and the Wider system, and they found that 47.1% of strains were considered resistant to methicillin.

### 3.4. Susceptibility to *β*-Lactam Antibiotics

**Figure 4:**
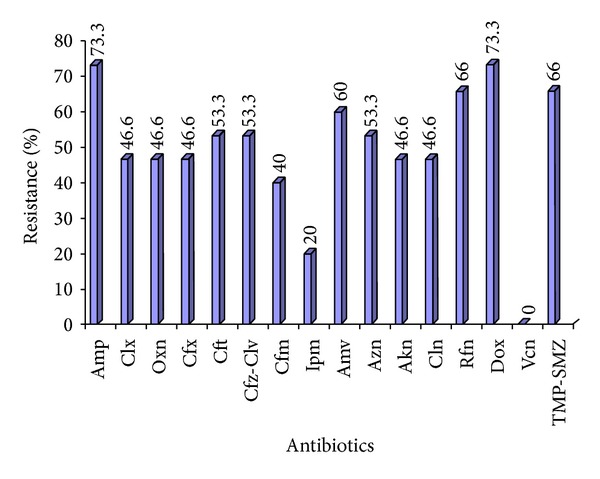
Percentages of antibiotic resistance among* Staphylococcus lugdunensis* isolates. Amp: ampicillin; Clx: cloxacillin; Oxn: oxacillin; Cfx: cefoxitin; Cft: ceftriaxone; Cfz-Clv: ceftazidime clavulunate; Cfm: cefixime; Ipm: imipenem; Amv: amoxiclav; Azn: azithromycin; Akn: amikacin; Cln: clindamycin; Rfn: rifampicin; Dox: doxycycline; Vcn: vancomycin; TMP-SMZ: trimethoprim/sulfamethoxazole.

The results revealed that 11 of 15* S. lugdunensis *isolates showed high resistance (73.3%) to ampicillin (Figure 4). Results also showed that the resistance rate to oxacillin and cloxacillin was 46.6%. Methicillin replaces methicillin as oxacillin which is stable under storage conditions, and methicillin actually is an excellent inducer of the* mecA* gene. Ezekiel and Adebayo [25] isolated three strains of* S. lugdunensis *of 149 CoNS; all isolates were resistant to oxacillin and other *β*-lactam antibiotics. Tan and his colleagues [22] in Singapore found that resistance to oxacillin was detected in 5% of the isolates.

Results of cefoxitin (2nd generation), ceftriaxone, and cefexime (3rd generation) showed that the percentages of* S. lugdunensis *resistant isolates were substantial to these antibiotics: 46.6%, 53.3%, and 40%, respectively (Figure 4). These results can be explained by the fact that all staphylococcal strains produce *β*-lactamase which destroys the *β*-lactam ring resulting in inactive products [26]. Tan and his colleagues [22] found that resistance to cefoxitin was detected in 5% of isolates.

The resistance rates to amoxiclav and ceftazidime-clavulanic acid were 60% and 53.3%, respectively. Clavulanic acid can inhibit the action of *β*-lactamase enzyme that causes decrease in the resistance of bacteria to *β*-lactam antibiotics [27]. Results found that* S. lugdunensis *isolates were susceptible to imipenem (80%). Imipenem inhibits bacterial cell wall synthesis by binding to and inactivating PBPs [28].

### 3.5. Susceptibility to Non-*β*-Lactam Antibiotics

Result of this study regarding susceptibility to amikacin found that the isolates showed low level of resistance (46.6%) to this antibiotic. The resistance rate of azithromycin was 53% (Figure 4). This resistance may be attributed to the efflux mechanism in staphylococci which is mediated by* MsrA*, a protein that induced by clarithromycin, azithromycin, and telithromycin and encoded by* msrA* gene [29]. Result of this study regarding susceptibility to clindamycin found that the isolates showed low level of resistance (46.6%) to this antibiotic. Hellbacher et al. [30] found 10% of* S. lugdunensis* isolates were resistant to clindamycin.* Staphylococcus lugdunensis *isolates results showed (73%) resistance to doxycycline. Tan and his colleagues [22] in Singapore found that resistance to tetracycline was 12% of isolates. The percentage of resistance for both trimethoprim-sulfamethoxazole and rifampin was 66%. Rifampin resistance in* Escherichia coli *and* S. aureus *is due to alterations in the target leading to a reduced affinity of the enzyme for the antibiotic [31].

### 3.6. Results of Antibiotic Resistance by MIC

In this study, 11 of 15 (73.3%)* S. lugdunensis *isolates were resistant to ampicillin (≥128 *μ*g/mL), while 4 of 15 were having MIC values reached 2 *μ*g/mL. The MIC values of* S. lugdunensis *isolates against oxacillin revealed that 5 of 15 isolates reached 32 *μ*g/mL, while MIC value of 2 isolates was ≥ 64 *μ*g/mL. Six oxacillin resistant isolates (S1, S2, S3, S4, S5, and S6) had* mecA *gene, but one isolate (S7) did not have such gene. Out of 15* S. lugdunensis *isolates (detected by MIC method), 14 isolates (93.2%) were sensitive to vancomycin, while only one isolate (6.8%) showed reduced susceptibility to vancomycin 8 *μ*g/mL (intermediate resistant). Bourgeois and his coworkers [31] found that 6/13* S. lugdunensis *isolates were tolerant to vancomycin. No isolates showed any degree of resistance to vancomycin as in many data. The* van*A genes that are responsible for resistance (*van *genes) are inducible and transferable and confer high-level resistance to vancomycin [32].

### 3.7. *β*-Lactamase Production

Eight isolates (53.3%) were *β*-lactamase producer. All these isolates were ampicillin resistant; seven of eight *β*-lactamase producing isolates were oxacillin resistant, while the remaining one was oxacillin sensitive. Six of eight were having* mecA *gene (Table 2).

Mateo and her coworkers [24] found that 11.8% of* S. lugdunensis *were *β*-lactamase producers. Several authors reported that the percentages of *β*-lactamase-positive* S. lugdunensis* vary from 24 to 40% in US isolates collections [33]. Papapetropoulos and his colleagues [34] isolated 14* S. lugdunensis *strains from clinical specimens (abscesses and wounds) of 250 bed-hospital in Athens, Greece; 5 (30.2%) of* S. lugdunensis *were *β*-lactamase positive. The difference between this study and other studies may be due to the fact that the global using of *β*-lactam antibiotics in Iraq may result in induction of bacterial resistance to *β*-lactams via production of *β*-lactamases [35].

### 3.8. Detection of* mecA *Gene in MRSL Isolates

**Figure 5:**
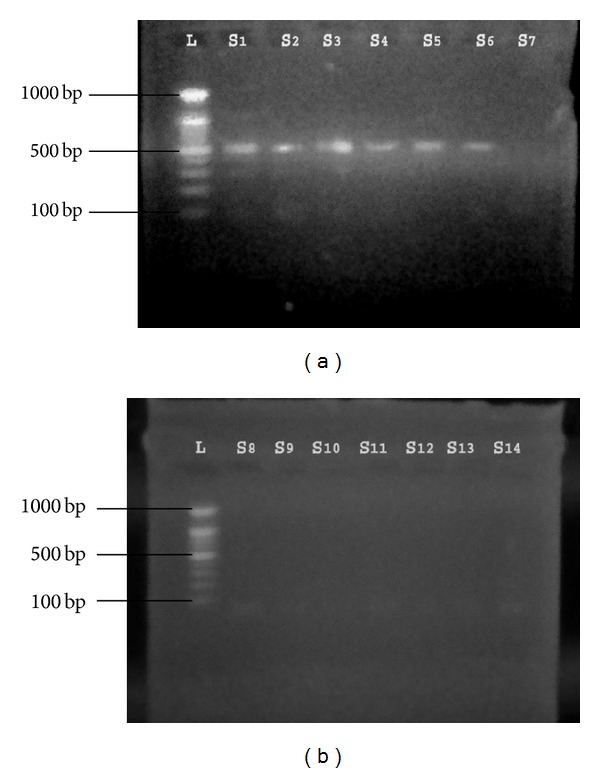
Gel electrophoresis of PCR of* mecA* amplicon (499 bp) product: Lane L: ladder (1000-bp ladder); Lanes S1, 2, 3, 4, 5, and 6:* mecA* positive; Lanes S7, 8, 9, 10, 11, 12, 13, and 14:* mecA* negative samples.

In this study* mecA *gene was detected in 6 (40%) of 15* S. lugdunensis *(Figure 5). All of these 6 isolates (S1, S2, S3, S4, S5, and S6) were resistant to oxacillin (Table 2). One isolate (S7) was resistant to oxacillin, but* mecA *was not detected in this isolates.

*Staphylococcus lugdunensis *is generally considered to be susceptible to oxacillin. Several studies reported negative PCR results when screening for* mecA*, but among reports in the English literature* mecA *has been detected in two* S. lugdunensis *isolates [8, 36]. Tee and his colleagues [8] reported a case of MRSL causing bloodstream infection in a neonate with an oxacillin MIC > 256 mg/L having* mecA *gene. In 2008, Tan and his colleagues [22] found five (4.7%)* S. lugdunensis *strains carrying the* mecA *gene in a collection of 106 clinical isolates.

### 3.9. Antibiotic Susceptibility and Detection of* mecA* Gene in* “S. pseudolugdunensis”* Isolates

The antibiotic susceptibility profile of* “S. pseudolugdunensis”* isolates (number = 7) is shown in Table 3. In addition to the results discussed earlier, this study represents the first record of characterization of oxacillin resistant and* mecA* positive* “S. pseudolugdunensis”* isolates recovered from clinical specimens in Iraqi hospitals (Table 4).

## 4. Conclusion

The highest percentages of* S. lugdunensis* isolates were recovered from subaxillary, wound swabs, and skin swabs samples, so this study reinforces the propensity of* S. lugdunensis *to be associated with acute cutaneous infections. Although many other reports stated that* mecA* gene presents low percentages in* S. lugdunensis*; however, the present study found high rate of* mecA* in these bacteria.

## Figures and Tables

**Table 1:** Numbers, sources, and percentages of coagulase negative staphylococci isolates.

Source	Number (%)	CoNS No. (%)
Wound swabs	105 (17.4)	42 (23.56)
Ear swabs	28 (4.6)	6 (3.37)
Urine swabs	50 (8.3)	11 (6.17)
Skin lesion swabs	25 (4.1)	10 ( 5.6)
Throat swabs	32 (5.3)	11 (6.17)
Burn swabs	80 (13.2)	15 (8.4)
Blood culture	69 (11.4)	13 (7.3)
Sputum	36 (5.9)	4 (2.24)
Sub axillary swabs∗	64 (10.6)	52 (29.2)
HVS∗∗	23 (8.3)	2 (1.12)
Stool swabs	38 (6.3)	2 (1.12)
Other	52 (8.6)	10 ( 5.6)

Total	602 (100)	178 (100)

*These samples were taken from immature patients in neonate intensive care unit.

**High vaginal swabs.

**Table 2:** Relationship between ampicillin, oxacillin resistance with presence of *mecA *gene, and *β*-lactamase production in *Staphylococcus lugdunensis *isolates.

Isolate number	Ampicillin resistant	Oxacillin resistant	mecA	*β*-Lactamase production
S1	+	+	+	+
S2	+	+	+	+
S3	+	+	+	+
S4	+	+	+	+
S5	+	+	+	+
S6	+	+	+	+
S7	+	+	−	+
S8	+	−	−	−
S9	+	−	−	−
S10	+	−	−	−
S11	+	−	−	+
S12	−	−	−	−
S13	−	−	−	−
S14	−	−	−	−
S15	−	−	−	−

**Table 3:** Antibiotic susceptibility profile of *“S. pseudolugdunensis”* isolates.

Isolate number	Ceftazidime-clavulanate	Ceftriaxone	Azithromycin	Imipenem	Doxycycline	amikacin	Cefixim	Amoxiclav	Vancomycin	Clindamycin	Rifampicin	TMP-SMZ
SP1	S	S	S	S	R	R	R	S	S	R	S	R
SP2	S	S	S	S	R	S	R	S	S	S	S	S
SP3	R	R	R	S	R	R	R	S	S	R	R	R
SP4	S	S	S	S	R	S	S	R	S	S	S	R
SP5	R	R	S	R	R	S	S	S	S	S	S	R
SP6	S	R	S	S	S	S	R	S	S	R	S	S
SP7	S	S	S	S	S	R	R	R	S	S	S	S

**Table 4:** Relationship between oxacillin resistance with presence of *mecA* gene and *β*-lactamase production in *“S. pseudolugdunensis”* isolates.

Isolate number	Resistance to				*β*-Lactamase production	*mecA *gene
	Ampicillin	Oxacillin 4 *μ*g/ml	Oxacillin 6 *μ*g/ml	Cefoxitin		
SP1	+	+	+	−	+	−
SP2	+	+	+	+	+	+
SP3	+	+	+	+	+	+
SP4	+	+	+	+	+	+
SP5	+	+	−	−	−	−
SP6	+	+	+	+	+	+
SP7	+	−	−	−	−	−
